# Why Should Clinical Autopsies Continue to Exist?

**DOI:** 10.3390/diagnostics11081482

**Published:** 2021-08-16

**Authors:** Simone Gusmão Ramos, Giulia Ottaviani, Luiz Cesar Peres, Bruna Amanda Cruz Rattis, Patricia Santos Leão, Thamiris Nadaf Akel, Leticia Ussem, Caio Antonio Campos Prado, Elaine Christine Dantas Moises, Lilian Christiane Andrade Grimm, Eliane Pedra Dias

**Affiliations:** 1 Department of Pathology and Forensic Medicine, Ribeirão Preto Medical School, University of São Paulo, Ribeirão Preto 14049-900, SP, Brazil; bruna.rattis@usp.br (B.A.C.R.); patricialeao@usp.br (P.S.L.); thamiris_nadaf@hotmail.com (T.N.A.); leticiaussem@gmail.com (L.U.); 2 Centro di Ricerca Lino Rossi, Anatomic Pathology MED-08, Università degli Studi di Milano, 20122 Milan, Italy; giulia.ottaviani@unimi.it; 3 Sheffield Children’s NHS Foundation Trust, Sheffield S10 2TH, UK; l.cesar.peres@gmail.com; 4 Department of Gynecology & Obstetrics, Women’s Health Reference Center of Ribeirão Preto (MATER), Ribeirão Preto Medical School, University of São Paulo, Ribeirão Preto 14090-900, SP, Brazil; cacprado@gmail.com (C.A.C.P.); elainemoises@fmrp.usp.br (E.C.D.M.); 5 Health Organization Management, Ribeirão Preto Medical School, University of São Paulo, Ribeirão Preto 14049-900, SP, Brazil; lilian.grimm@hotmail.com; 6 Department of Pathology, Faculty of Medicine, Fluminense Federal University, Niteroi 24220-900, RJ, Brazil; elianepedradias@gmail.com

## 1. Introduction

At some point in history, medicine was integrated with pathology, more precisely, with pathological anatomy. With the commencement of medical autopsies, the concept of anatomical–clinical correlation put forth by Giovanni Battista Morgagni (1682–1771) started unraveling the mysteries of cause and effect in the bodies dissected in the anatomy amphitheater of the University of Padua. Morgagni and his colleagues discovered the correlation between the changes in the tissues observed in cadaver organs and the patients’ symptoms. His famous book, *De sedibus et causis morborum per anatomen indagatis*, published in 1761, is still referenced in medical schools [1]. This method, however, was established as a specific discipline called pathological anatomy almost a century later through the works of Karl Von Rokitansky (1804–1878) at the Vienna Institute of Pathological Anatomy [2].

Unfortunately, acknowledgment of the importance of the autopsy room as a place of learning, teaching, and research lasted only about 200 years. After peaking in the mid-20th century, clinical autopsies comprised 50% of all autopsies performed at most academic medical centers, and this ratio has now reduced to 4%–8% [3]. The reasons for the decrease in the rates of the autopsy are multifactorial and complex [4,5,6,7]. Despite this sharp decline, clinical autopsies continue to be essential for medical diagnosis in some select areas of medicine. This is especially true in areas that are less accessible clinically (e.g., cardiopathology, neuropathology) or even those areas in which a well-founded clinical diagnosis is unlikely to be achieved without a well-performed autopsy (e.g., sudden death of an adult or infant, fetal death due to genetic alterations).

Autopsies provide a wealth of information that can aid in significant and decisive advancements in improving people’s quality of life. However, these days, it is necessary to align the practice of autopsy examination with the current needs of the healthcare system to improve medical practice, medical education, medical research, government, and public health sectors. Therefore, this special issue about autopsy aimed to highlight the fundamental role of traditional clinical autopsy in medical practice and to discuss how postmortem examination can be adapted to the current times so that it continues to contribute to the progress of medical science.

## 2. The Importance of Clinical Autopsy in Medical Training

The pathology needs to be part of a medical student’s daily life, including autopsy. In-depth and detailed knowledge of the patient’s body is essential in all phases of medical practice. Autopsies present a learning opportunity where one can observe the limits of normality in the organs, deepen the understanding of incipient or even asymptomatic lesions, recognize diseases through the body’s responses, understand human aging, and, finally, accurately establish the mechanism and the cause of death. The task of including the autopsy room in the daily teaching-learning-evaluation of medical education is not easy. However, having a well-equipped autopsy room available is a good start.

Students should visit the autopsy rooms as a cross-curricular activity during medical school. Learning different topics, including anatomy, physiology, histology, genetics, embryology, and immunology, can be highly engaging by participation in autopsies. If feasible, the autopsy rooms should be attended by groups of students from different years of medical school, where the activities and the responsibilities would be different but integrated. In this context, the pathologist could be the physician performing the intersection and the integration between students and the other medical teams involved, including imaging and laboratory tests. The triad—patient care, diagnostic technology, and clinical autopsy—is the most appropriate context for acquiring medical skills. This can offer unparalleled knowledge and a learning workshop to young aspiring physicians, promoting the concept of a holistic view of the patient from an early age and integrating the pillars of medical knowledge offered in universities: teaching, research, and assistance.

## 3. The Need for Autopsy in the Training of Pathologists

In 1936, with the foundation of the American Pathological Association, the certification of pathologists was initiated. The standardization of training in the area promoted the improvement in postmortem examination techniques. Autopsied cases provide a unique opportunity for the novice pathologist to correlate the information from physical examination, laboratory tests, and imaging exams with the changes identified in the cadaver. When the beginning resident has most of his work inside the autopsy room, he develops a broader anatomic-clinical view. It is possible to learn, through each case, the main macroscopic and the microscopic findings of several diseases, thus adding a greater value to their learning. Therefore, the autopsy provides fundamental data for improving the quality of medical training and care.

At a 2014 meeting of the Association of Pathology Chairs (APC), some pathologists suggested discontinuing autopsy from the pathology resident training curriculum to provide additional months for training in new disciplines such as molecular genetics and informatics [8]. At the same time, the American Board of Pathology (ABP) received complaints that newly hired pathologists with recent certificates in pathological anatomy were not adequately skilled at performing autopsies when requested. In response to the call to discontinue autopsy from pathology training on the one hand and the call for more rigorous autopsy training on the other, the APC formed the Autopsy Working Group to examine the role of autopsy in pathology residency training. In 2016, the Autopsy Working Group submitted its final report to the APC. All members of the Autopsy Working Group agreed that autopsy training is an essential component in transforming a newly graduated physician into a competent anatomical pathologist [8].

## 4. Autopsies of Fetuses with Genetic Alterations

Fetal losses in the first trimester and the early second trimester of gestation provide the opportunity for fetal autopsy. This is particularly relevant in pregnancies of up to 15 weeks, which sometimes need to be performed with the aid of tools, such as magnifying glasses and dissection microscopes, in addition to delicate instruments, such as those used in ophthalmologic surgeries [9]. Fetuses and placentas examination should ideally be done in the fresh state prior to fixation. In addition to facilitating dissection, this condition is ideal for collecting samples for microbiological culture or skin/placenta for genetic testing. Most fetal losses during this period are chromosomal in origin. In addition to a detailed macroscopic examination, it is necessary to collect material for the histopathological study of the major organs. Some changes can be identified even in partially autolyzed tissue. Of note, the fetuses may be always photographed and radiographed before the start of the examination.

By definition, genetic diseases are congenital, although their expression might manifest much later after birth, sometimes in adulthood. However, those that manifest early, usually during embryogenesis, tend to be found more frequently in the autopsy room. It is important to reiterate that not all congenital anomalies are genetic in origin, as they might result from errors of morphogenesis or cellular injury by environmental factors, including physical (such as ionizing radiation, fever), chemical (therapeutic drugs or not), infectious (such as cytomegalovirus, herpes, toxoplasmosis, syphilis, Zika virus), and unknown (diaphragmatic hernia) causes. The correct identification of anomalies and the subsequent genetic investigation are essential for appropriate reproductive counseling, thereby reassuring the families who would otherwise feel helpless.

## 5. Clinical Autopsy on Maternal Death

For planning and implementation of strategies with a significant impact on the reduction of maternal mortality (MM), it is essential to characterize and understand the main causes of MM. They are mainly: hemorrhage (27%), hypertensive diseases (14%), sepsis (11%), abortions (8%), and embolia (3%) [10]. There are also indirect causes of MM due to pre-existing health conditions. However, the list of leading causes shows that many deaths can be avoided if identified and treated early. Unfortunately, it occurs in about 60% of cases of maternal death [11].

Maternal Mortality Review Committees serve as effective strategies to reduce maternal deaths from preventable causes. These are multidisciplinary groups that seek multiple sources of information to revisit the history of each death event using patient medical records at all points of care in the hierarchy of care, prenatal care cards, statements from professionals, death certificates, interviews with family members (verbal autopsy), police reports, and necropsy reports. Analyzing these data allows the committee members to establish the chronological sequence and the possible relationships between the events that led to death, to define the cause of death if related to pregnancy, and to identify whether it was a preventable death. Since these data comprise all levels and care settings, it is possible to identify gaps in the line of care, even if not related to the death itself [12].

One of the significant challenges for the Maternal Mortality Review Committees is when clinical data are scarce or inadequate to arrive at an appropriate diagnostic correlation. Real cases that are not infrequent during the evaluations can be exemplified, such as situations in which a pregnant woman is found to have died at home or in cases that arrive at the emergency room in extremely serious conditions, leading to death in a short time. In such circumstances, a structured autopsy is crucial for identification of the cause of death in approximately 80% of such cases and, consequently, a proper definition of action plans to be proposed [13]. It is also worth mentioning that some clinical situations might not be suspected during the patient’s evaluation, even with good prenatal and hospital care, such as congenital heart disease, myocarditis, cardiomyopathy, amniotic embolism, and some primary pulmonary diseases. In this context, performing a systematic clinical autopsy is important to identify the causes of direct or indirect maternal death to assess the possibility that death was preventable and, consequently, define public health strategies.

## 6. Clinical Autopsy in Sudden Death

Sudden unexpected death (SUD) is a fatal event that occurs in an apparently healthy individual, such that an abrupt outcome could not have been predicted. SUD, by definition, is sudden and occurs as the first manifestation of an unknown underlying disease or within a few hours of presentation of a disease and is unexpected from the medical history. SUDs—including sudden unexpected death of the young (SUDY), sudden infant death syndrome (SIDS), and sudden intrauterine unexplained death (SIUD)—are the major unexplained, distressing form of death that can occur. It can happen at any time without warning. SUD represents a significant public health problem with an overwhelming economic and emotional impact on the involved families and communities [14].

Environmental risk factors and triggers can interact in complex ways with the patients’ genetic history to affect the nervous and the cardiac autonomic conduction systems resulting in SUD; however, an accurate examination of these anatomical structures remains neglected. Recent studies showing abnormalities in the cardiac conduction system and the brainstem regulatory nuclei in SIDS-SIUD [15,16] point to evidence to warrant a detailed postmortem examination in all cases of SUD at any age. Such an approach includes the close examination of the cardiac conduction system, where the electrical impulse starts, and of the brainstem nuclei that regulate circulatory, respiratory, and arousal activities of the human body according to the investigation protocol elaborated upon by the Lino Rossi Research Center, Università degli Studi di Milano [17]. The morphological bases of SUD, defined as *sine materia*, are most accurate when examined by personnel trained in the autonomic and the conduction systems of the heart. Critical pathological findings help to provide information to surviving family members, especially about genetic abnormalities, as well as to make correlations with exposure to environmental risk factors and to identify potential triggers that might interact with genetic predisposition, showing, eventually, intriguing overlaps.

## 7. The Future of Clinical Autopsy

Despite the decrease in the rate of postmortem examination, autopsy remains the gold standard for determining how and why an individual died. This major role was supported by several studies demonstrating that, even with accurate diagnostic techniques and intensive patient monitoring, the principal diagnosis of death was not correctly identified in about 30% of the cases [18,19]. Nevertheless, there is no doubt about the need for developing new practices that are more in line with the current status of medicine and agree with the discussion on the future of autopsies in current years.

One promising alternative is computed tomography (CT) images as an adjuvant in diagnosing the cause of death during an autopsy, a practice known as virtopsy [20]. Although still in its first decade, it is already clear that autopsy and CT are complementary examination modalities [21]. Images of fluid collections, hypertensive pneumothorax, cerebral hemorrhage, and even an acute aortic dissection followed by cardiac tamponade can be observed in the cadaver. In forensic autopsy, its value in defining fractures in patients with polytrauma or even in the localization of a bullet projectile is unquestionable. This model of postmortem examination represents a promising future in which pathologists and radiologists can work together as specialists in diagnosing deaths that occurred without medical assistance, avoiding the entire autopsy procedure.

With the advent of “personalized medicine”, especially in treating patients with cancer, pathology assumed a crucial role in determining cancer therapies with specific molecular guidance for mutated genes. An excellent option increasingly used is the minimally invasive autopsy (MIA) guided by imaging examinations [22]. MIA would play a preponderant role in clinical research with the targeted retrieval of tumor fragments from metastases or recurrences after aggressive treatments such as chemotherapy or radiotherapy. We could follow the “evolution” of tumor mutations and better understand tumor biology in vivo. In addition to being more economical than the conventional autopsy, it can be performed in a shorter time after death, and, eventually, it can be adapted to be performed at the bedside of a recently deceased patient. Moreover, it is less invasive to the body, which may facilitate easier consent of the family members.

Despite limitations inherent to the procedure, MIA might be decisive in specific cases, such as confirmation of disseminated malignant neoplasia, direct study of organs affected in diseases, or when there exists a high risk of infection for the team involved [23], among others. The fact that it can be performed in a shorter duration post mortem makes it feasible to obtain material that can be frozen for use in more sophisticated techniques and methodologies of investigation involving molecular and genetic pathology. Even with the advancement of technology and that more sophisticated techniques can currently be performed on paraffined material [4], the reduced postmortem time and the freezing of the tissues obtained would undoubtedly increase the accuracy of these diagnoses. This would also open new opportunities for pathologists to pursue advanced studies from materials obtained during the clinical practice of pathology, including autopsy.

In recent times, advances in basic medical science and imaging seemed to reduce the need for autopsies. However, the current global pandemic has revealed just the opposite. At this time of the coronavirus disease 2019 (COVID-19) pandemic, and with fewer autopsies performed thus far, the world is struggling to treat a disease whose pathological basis is unknown. With a huge number of patients contracting the disease simultaneously, there is a broad spectrum of different aspects of the same disease, which has made it even more challenging to understand and predict the course of the disease. Despite tens of thousands of scientific papers published by different researchers, the puzzle remains unresolved. This experience demonstrates the current distance between basic science and clinical science [24]. Autopsy could play a fundamental role in successfully addressing complex human disease issues in an integrated manner. Herein, the lockdown was of the science [25,26,27].

Research in animal models and in vitro cells enables the understanding of cell functions, molecules, and their pathways of action and ensures technological advances; however, no research method reproduces the complexity and the specificities of actual disease in the patient as the autopsy does. Indeed, a completely distorted and calcified coronary tree does not necessarily indicate the cause of death as acute myocardial infarction. It indicates that the patient had a severe ischemic atherosclerotic heart disease as the underlying cause of disease. In fact, in order to avoid garbage codes (GC; Ill—defined and unknown causes of mortality), autopsy pathologists now should direct their diagnoses to the underlying cause compatible with the clinical picture that culminated in the death of the individual. This is currently adequate for the epidemiological data of public health. However, the immediate cause of death—an acute myocardial infarction with an occlusive thrombus in an unstable atherosclerotic plaque as well as the corresponding area of coagulative necrosis in the myocardium—whether there was reperfusion or not, the peri-infarct area, and others currently seem to be exclusively for academic interest.

Finally, it is important to remember that some tissue types are rarely available through means other than autopsy. In the case mentioned above, accurate data on the pathobiology of atherosclerotic plaques, including their behavior after angioplasty or coronary artery bypass graft (CABG), in addition to the efficacy of the recommended treatment show that all this information would definitely point to effective advances in the treatment of coronary ischemic disease. Unfortunately, precious information is being lost. Therefore, autopsy can still play a crucial role in the assessment of clinical outcomes in addition to always being a valuable source of material for scientific research [28].

## 8. Conclusions

(a) Historically, an autopsy always fulfilled various purposes, including those pertaining to medical care (diagnostic-related groups, quality assurance, and total patient care), medical science body (research, education), society (public health, vital statistics, forensic issues), and the family (counseling and understanding the life cycle).
(b) The participation of medical students in autopsy rooms during graduation is crucial to arouse the young physicians’ interest in pathological anatomy and promote early understanding of the patient with a holistic view.
(c) Autopsy is essential for the excellent training of pathologists. A strong foundation in autopsy practice catalyzes the transformation of a medical student into a practicing pathologist who can evaluate the data in a given case and synthesize this information to provide the correct diagnosis. Moreover, it is often the only opportunity to study and analyze organs and tissues that are challenging to access in daily medical practice.
(d) Fetal/infant autopsy, whether in cases of genetic changes or sudden death, besides the scientific aspect, has a crucial social connotation in helping the victimized families overcome their mourning.
(e) A complete autopsy study is crucial to define unclear causes of maternal death.
(f) Sudden death, especially in young patients, can be clarified after detailed study of the conduction system and the regulatory centers of cardiac and/or respiratory activities in the brainstem or through the discovery of genetic diseases confirmed using molecular biology techniques.
(g) Despite the abundance of modern clinical and imaging tests, autopsies still improve the integrity and the reliability of national mortality data on which health strategies may be based.
(h) In the current times, autopsies could help to understand many remaining questions about the pathogenesis and the cause of death in patients with COVID-19.
(i) Implementing new contemporary approaches to autopsy (e.g., virtopsy, MIA, molecular pathology) can lead to an unexpected renaissance of the autopsy, leading to improved quality of health care delivery, teaching, and research.
(j) Autopsies can provide precious material for advancing medical research, providing a unique opportunity to study the pathogenesis of different disease processes and their influence on other organs in the human body.
(k) Pathologists must reform autopsy to make it relevant to current practice. Pathologists must fight for the survival of autopsies.

## Data Availability

Data sharing not applicable.
